# Protonation of γ‐Butyrolactone and γ‐Butyrolactam

**DOI:** 10.1002/open.202000220

**Published:** 2020-12-17

**Authors:** Stefanie Beck, Michael Feller, Laura Spies, Kai J. Dietrich, Christoph Jessen, Karin Stierstorfer, Andreas J. Kornath

**Affiliations:** ^1^ Department of Chemistry Ludwig-Maximilian University of Munich Butenandtstr. 5–13 81377 München Germany

**Keywords:** γ-butyrolactone, γ-butyrolactam, interatomic contacts, mapped electrostatic potentials, superacidic systems

## Abstract

**Abstract**: γ‐Butyrolactone and γ‐butyrolactam were reacted in the superacidic systems *X*F/*M*F_5_ (*X*=H, D; *M*=As, Sb). Salts of the monoprotonated species of γ‐butyrolactone were obtained in terms of [(CH_2_)_3_OCOH]^+^[AsF_6_]^−^, [(CH_2_)_3_OCOH]^+^[SbF_6_]^−^ and [(CH_2_)_3_OCOD]^+^[AsF_6_]^−^ and the analogous lactam salts in terms of [(CH_2_)_3_NHCOH]^+^[AsF_6_]^−^, [(CH_2_)_3_NHCOH]^+^[SbF_6_]^−^ and [(CH_2_)_3_NDCOD]^+^[AsF_6_]^−^. The salts were characterized by low temperature Raman and infrared spectroscopy and for both protonated hexafluoridoarsenates, [(CH_2_)_3_OCOH]^+^[AsF_6_]^−^ and [(CH_2_)_3_NHCOH]^+^[AsF_6_]^−^, single‐crystal X‐ray structure analyses were conducted. In addition to the experimental results, quantum chemical calculations were performed on the B3LYP/aug‐cc‐pVTZ level of theory. As in both crystal structures C⋅⋅⋅F contacts were observed, the nature of these contacts is discussed with Mapped Electrostatic Potential as a rate of strength.

## Graphical abstract

**The protonation of** γ‐butyrolactone and γ‐butyrolactam in the superacidic media HF/*M*F_5_ (*M*=As, Sb) occurs exclusively on the respective carbonyl oxygen atoms and leads to the formation of a π‐hole on the respective sp^2^‐hybridized carbon atoms. Surprisingly, the protonated species display C⋅⋅⋅F contacts in the solid state. The nature of these contacts is discussed on the basis of quantum chemical calculations.

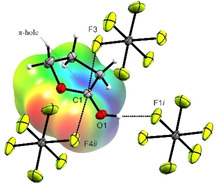

## 1 Introduction

Many natural products include a γ‐butyrolactone or γ‐butyrolactam moiety as structural element.[^1^, ^2^] Especially the γ‐butyrolactone motif is present in about 10 % of all natural products.[^3^, ^4^, ^5^] In total syntheses, a common strategy to synthesize the γ‐butyrolactone motif is the Baeyer‐Villinger oxidation.[^2^, ^6^] Herein, an ester is formed from a ketone by using peroxyacids or peroxides. The Beckmann rearrangement instead, which is used to obtain γ‐butyrolactam motifs, is the rearrangement of an oxime into an amide under ring expansion.[^2^, ^7^] Another synthesis strategy for both cyclic compounds is the catalyzed cyclodehydration of the corresponding hydroxyl acid,^[8]^ respectively amino acid.^[9]^ Interestingly, in case of γ‐butyrolactone, both, the lactonization^[8]^ and the hydrolysis,^[10]^ are acid‐catalyzed (see Equation 1.
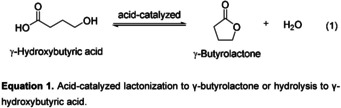



Since both cyclic compounds are abundant in natural products and their protonated species can occur in syntheses or metabolic cycles, they are of great interest. However, so far, only NMR‐spectroscopic investigations of protonated γ‐butyrolactone and protonated γ‐butyrolactam are reported.[^11^, ^12^] Under acidic conditions, a ring opening reaction can occur, which prompted us to investigate the reaction behavior of both compounds in superacidic media.

## 2 Results and Discussion

### 2.1 Preparation

The synthesis of salts of monoprotonated γ‐butyrolactone and γ‐butyrolactam was performed according to Equations. (2) and 3

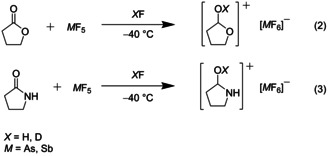



Hydrogen or deuterium fluoride, which serve as reagent as well as solvent, were used in excess for preparing the superacidic system. The respective Lewis acids were added, and the resulting mixture was homogenized at −40 °C. After cooling to −196 °C, the starting material γ‐butyrolactone, respectively, γ‐butyrolactam, was added to the frozen system under nitrogen atmosphere. After warming to −40 °C, all reactants were mixed to form the protonated species, respectively the deuterated species, and the excess of the solvent was removed in a dynamic vacuum at −78 °C. The air‐ and temperature‐sensitive compounds of monoprotonated γ‐butyrolactone [(CH_2_)_3_OCOH]^+^[AsF_6_]^−^ (**1**), [(CH_2_)_3_OCOH]^+^[SbF_6_]^−^ (**2**) and the deuterated species [(CH_2_)_3_OCOD]^+^[AsF_6_]^−^ (**3**) were obtained as colorless solids, which decompose at −30 °C. The salts of monoprotonated γ‐butyrolactam were obtained in terms of [(CH_2_)_3_NHCOH]^+^[AsF_6_]^−^ (**4**) and [(CH_2_)_3_NHCOH]^+^[SbF_6_]^−^ (**5**). Using the superacidic system DF/AsF_5_ led to an H/D exchange at the amide group. The hydrogen atoms were entirely replaced by deuterium and the degree of deuteration reached approximately 98 %. The deuterated species is observed in the form of [(CH_2_)_3_NDCOD]^+^[AsF_6_]^−^ (**6**). Salts (**4**), (**5**) and (**6**) are stable up to room temperature. With a larger ratio of Lewis acid, neither for γ‐butyrolactone nor for γ‐butyrolactam, a diprotonated species was observed.

### 2.2 Vibrational Spectroscopy

#### 2.2.1 Protonated γ‐Butyrolactone

**Figure 1:**
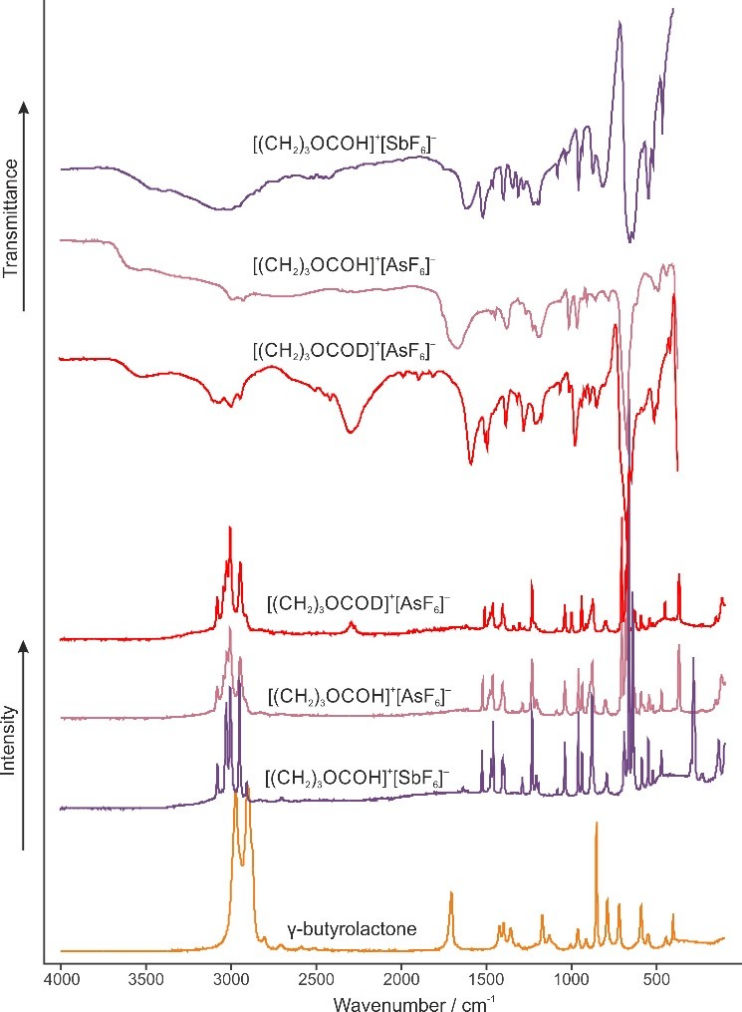
Raman spectrum of γ‐butyrolactone, infrared and Raman spectra of [(CH_2_)_3_OCOH]^+^[AsF_6_]^−^ (**1**), [(CH_2_)_3_OCOH]^+^[SbF_6_]^−^ (**2**) and [(CH_2_)_3_OCOD]^+^[AsF_6_]^−^ (**3**).

In Figure 1, the Raman and infrared spectra of [(CH_2_)_3_OCOH]^+^[AsF_6_]^−^ (**1**), [(CH_2_)_3_OCOH]^+^[SbF_6_]^−^ (**2**) and [(CH_2_)_3_OCOD]^+^[AsF_6_]^−^ (**3**) are displayed together with the Raman spectrum of γ‐butyrolactone. Selected experimental frequencies of (**1**), (**2**) and (**3**) together with quantum‐chemically calculated frequencies of the free cations [(CH_2_)_3_OCOH]^+^ and [(CH_2_)_3_OCOD]^+^ are summarized in Table 1. In Table S1, all observed and calculated frequencies are listed (see Supporting Information). In consequence of the *C*_1_ symmetry of the starting material,^[13]^ no higher symmetry, such as the ideal *C*_s_, is expected for the protonated species. We assumed *C*_1_ symmetry with 33 fundamental vibrations, active in Raman and infrared spectra, for the cation.

**Table 1:** Selected experimental vibrational frequencies [cm^−1^] of (**1**), (**2**) and (**3**), and calculated vibrational frequencies [cm^−1^] of [(CH_2_)_3_OCOH]^+^ and [(CH_2_)_3_OCOD]^+^.

[(CH_2_)_3_OCOH]^+^[AsF_6_]^−^ (1)		[(CH_2_)_3_OCOH]^+^[SbF_6_]^−^ (2)		[(CH_2_)_3_OCOD]^+^[AsF_6_]^−^ (3)		[(CH_2_)_3_OCOH]^+^	[(CH_2_)_3_OCOD]^+^	Assignment^[b]^
IR	Raman	IR	Raman	IR	Raman	Calc. (IR/Ra)^[a]^	Calc. (IR/Ra)^[a]^	
3528 (vw, br)		3460 (vw, br)		2307 (m)	2293 (14)	3724 (256/61)	2714 (148/29)	ν(O*X*)
1684 (s)	1613 (7)	1616 (m)	1637 (5)	1607 (s)	1619 (12)	1650 (305/0.7)	1635 (383/0.5)	ν(CO)
	1523 (24)	1522 (m)	1526 (14)	1510 (m)	1510 (27)	1523 (161/4)	1515 (74/8)	ν(CO)
1088 (m)	1089 (5)	1084 (w)	1085 (5)	912 (m)	916 (13)	1178 (199/7)	863 (12/1)	δ(CO*X*)
989 (m)		1012 (w)		960 (m)	961 (7)	1029 (3/3)	1029 (6/3)	ν(CC)
955 (m)	959 (33)	959 (m)	961 (24)	1001 (s)	1000 (21)	949 (5/4)	993 (107/6)	ν(CO)
932 (m)	938 (23)	937 (w)	939 (13)	939(m)	941 (35)	934 (1/3)	934 (2/4)	ν(CC)
881 (m)	877 (38)	876 (m)	878 (28)	876 (m)	875 (33)	881 (17/6)	889 (45/5)	ring breathing
806 (m)	800 (13)		793 (8)	800 (m)	799 (15)	796 (11/2)	796 (10/2)	δ(COC)
644 (w)	632 (21)	639 (vs)	633 (50)		450 (30)	611 (77/3)	443 (57/0.2)	δ(COX)_oop_
515 (m)	525 (9)	525 (s)	524 (3)	523 (m)	523 (13)	517 (25/0.5)	535 (1/0.7)	γ(CCOO)

[a] Calculated on the B3LYP/aug‐cc‐pVTZ level of theory. IR intensity in km/mol and Raman intensity in Å^4^/u. Abbreviations for IR intensities: v=very, s=strong, m=medium, w=weak, br=broad. Experimental Raman activities are stated to a scale of 1 to 100. [b] *X*=H, D.

A first evidence for a successful protonation is given by the ν(OH) vibration in the infrared spectra of (**1**) and (**2**), which occurs at 3528 cm^−1^ (**1**) and 3460 cm^−1^ (**2**), respectively. In the corresponding Raman spectra, no lines are observed, because of the poor polarizability of the OH stretching vibration. Contrariwise, the OD stretching vibration of [(CH_2_)_3_OCOD]^+^[AsF_6_]^−^ (**3**) is detected at 2239 cm^−1^ (Ra) and 2307 cm^−1^ (IR). The redshift is in good agreement with the Teller‐Redlich rule for an H/D isotopic effect.^[14]^ Considering the constitution of the synthesized cation, the ring breathing vibration at about 880 cm^−1^ (Ra, IR) indicates the preservation of the ring structure. Changes in vibrational spectra are observed for the CO stretching vibrations. Due to the protonation, the former CO double bond is weakened and the ν(CO) occurs at 1523 cm^−1^ (Ra) (**1**), at 1522 cm^−1^ (IR) and 1526 cm^−1^ (Ra) (**2**) and at 1510 cm^−1^ (IR and Ra) (**3**).

Compared to the neutral compound,^[15]^ this redshift amounts up to 260 cm^−1^. In contrast, the former CO single bond, belonging to the lactone moiety, is strengthened and the corresponding stretching vibration is detected at 1684 cm^−1^ (IR) and 1613 cm^−1^ (Ra) (**1**), at 1616 cm^−1^ (IR) and 1637 cm^−1^ (Ra) (**2**) and at 1607 cm^−1^ (IR) and 1619 cm^−1^ (Ra) (**3**). The blue shift is up to 306 cm^−1^ compared to γ‐butyrolactone.^[15]^ The other CO stretching vibration of the ring skeleton is detected at about 960 cm^−1^ (IR, Ra) for the protonated species (**1** and **2**) and at about 1000 cm^−1^ for the deuterated one (**3**). The δ(COH), respectively the δ(COD), is observed in the range between 1084 cm^−1^ and 1089 cm^−1^ for (**1**) and (**2**) and at 912 cm^−1^ (IR) and 916 cm^−1^ (Ra) for (**3**). More vibrations, which are assigned to the AsF_6_^−^, respectively the SbF_6_^−^ anion, are observed than were expected for an ideal *O*_h_ symmetry. Here, in the Raman spectra more than three lines and in IR spectra more than two bands are detected. The increased number of vibrations indicates a lowered symmetry of the structure of the anions.

#### 2.2.2 Protonated γ‐Butyrolactam

**Figure 2:**
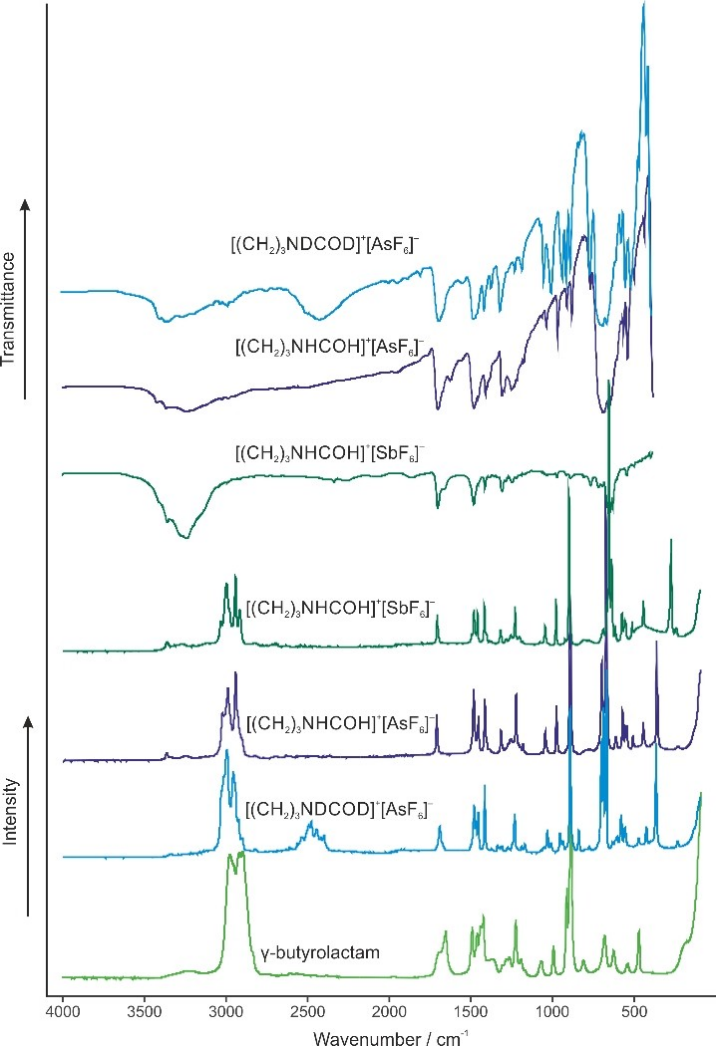
Raman spectrum of γ‐butyrolactam, infrared and Raman spectra of [(CH_2_)_3_NHCOH]^+^[AsF_6_]^−^ (**4**), [(CH_2_)_3_NHCOH]^+^[SbF_6_]^−^ (**5**) and [(CH_2_)_3_NDCOD]^+^[AsF_6_]^−^ (**6**).

The Raman spectrum of γ‐butyrolactam together with Raman and IR spectra of [(CH_2_)_3_NHCOH]^+^[AsF_6_]^−^ (**4**), [(CH_2_)_3_NHCOH]^+^[SbF_6_]^−^ (**5**) and [(CH_2_)_3_NDCOD]^+^[AsF_6_]^−^ (**6**) are illustrated in Figure 2. In Table 2, selected experimental frequencies of (**4**), (**5**), and (**6**) with calculated frequencies of the free cations [(CH_2_)_3_NHCOH]^+^ and [(CH_2_)_3_NDCOD]^+^ are summarized. All observed and calculated frequencies are listed in Table S2 (see Supporting Information).

**Table 2:** Selected experimental vibrational frequencies [cm^−1^] of (**4**), (**5**) and (**6**), and calculated vibrational frequencies [cm^−1^] of [(CH_2_)_3_NHCOH]^+^ and [(CH_2_)_3_NDCOD]^+^.

[(CH_2_)_3_NHCOH]^+^[AsF_6_]^−^ (4)		[(CH_2_)_3_NHCOH]^+^[SbF_6_]^−^ (5)		[(CH_2_)_3_NDCOD]^+^[AsF_6_]^−^ (6)		[(CH_2_)_3_NHCOH]^+^	[(CH_2_)_3_NDCOD]^+^	Assignment^[b]^
IR	Raman	IR	Raman	IR	Raman	Calc. (IR/Ra)^[a]^	Calc. (IR/Ra)^[a]^	
3238 (w)		3242 (vs)		2492 (w)	2494 (18)	3750 (235/67)	2732 (137/33)	ν(O*X*)
3364 (w)	3369 (3)	3358 (s)	3359 (4)	2430 (w)	2445 (16)	3553 (139/82)	2609 (89/37)	ν(N*X*)
1711 (m)	1716 (16)	1711 (m)	1711 (14)	1691 (m)	1691 (17)	1729 (251/3)	1703 (278/5)	ν(CN)
1462 (w)	1461 (16)	1464 (vw)	1464 (14)	1462 (m)	1456 (24)	1500 (103/7)	1474 (140/5)	ν(CO)
1390 (w)	1393 (4)	1393 (vw)		1186 (w)	1172 (7)	1404 (3/0.7)	1176 (6/2)	δ(CN*X*)
1186 (w)	1189 (5)		1189 (4)	954 (w, sh)	956 (13)	1198 (138/8)	915 (28/9)	δ(CO*X*)
1049 (w)	1052 (10)		1048 (9)	1055 (m)	1056 (6)	1042 (2/2)	1032 (0.3/3)	ν(CN)
982 (m)	983 (20)	982 (vw)	982 (20)	943 (m)	938 (9)	971 (10/3)	938 (11/5)	ν(CC)
926 (w)	924 (sh)	928 (vw)	928 (5)	775 (s)	778 (6)	929 (3/0.2)	809 (6/0.4)	ν(CC)
895 (w)	897 (73)	903 (vw)	902 (58)	895 (m)	895 (79)	896 (5/17)	889 (52/8)	ring breathing
517 (m)	518 (9)	515 (vw)	517 (11)	559 (s)	560 (16)	497 (24/0.6)	665 (21/1)	γ(CCON)

[a] Calculated on the B3LYP/aug‐cc‐pVTZ level of theory. IR intensity in km/mol and Raman intensity in Å^4^/u. Abbreviations for IR intensities: v=very, s=strong, m=medium, w=weak. Experimental Raman activities are stated to a scale of 1 to 100. [b] *X*=H, D.

For protonated γ‐butyrolactam, *C*_1_ symmetry with 36 IR and Raman active vibrations, is expected. The assumption of retaining the ring structure is confirmed by the ring breathing vibration at about 900 cm^−1^ (Ra, IR).

The OH stretching vibration, which is the first evidence for a successful protonation, is observed in the IR spectra at 3238 cm^−1^ (**4**) and 3242 cm^−1^ (**5**). In the corresponding Raman spectra, these lines are not observable, due to the poor polarizability of this vibration. In contrast, the ν(NH) vibration occurs in both IR (3364 cm^−1^ (**4**) and 3358 cm^−1^ (**5**)) and Raman spectra (3369 cm^−1^ (**4**) and 3359 cm^−1^ (**5**)). In the spectra of the deuterated species (**6**), the OD stretching vibration occurs at 2492 cm^−1^ (IR) and 2494 cm^−1^ (Ra). The ν(ND) is observed at 2430 cm^−1^ (IR) and 2445 cm^−1^ (Ra). These redshifts are in accordance with the Teller‐Redlich rule for an H/D isotopic effect.^[14]^ The δ(CO*X*) are observed at about 1190 cm^−1^ for the protonated species (**4** and **5**) and at about 955 cm^−1^ for the deuterated species (**6**). δ(CN*X*) occurs at about 1390 cm^−1^ (**4** and **5**) and at 1180 cm^−1^ (**6**). Due to the protonation, the CO bond is weakened and the vibration occurs at 1462 cm^−1^ (IR) and 1461 cm^−1^ (Ra) (**4**), 1464 cm^−1^ (IR, Ra) (**5**), and at 1462 cm^−1^ (IR) and 1456 cm^−1^(Ra) (**6**). Compared to the neutral compound, this vibration is redshifted by up to 194 cm^−1^.^[15]^ Contrariwise, the CN bond is strengthened and its stretching vibration is observed at 1711 cm^−1^ (IR (**4**) and IR and Ra (**5**)), 1716 cm^−1^ (Ra) (**4**) and 1691 cm^−1^ (IR, Ra) (**6**). This blue shift amounts up to 222 cm^−1^ compared to γ‐butyrolactam.^[15]^ The remaining stretching modes for the ring bonds nearly are unaffected by the protonation.

For the anions with ideal *O*_h_ symmetry, three lines in Raman and two bands in IR spectra are expected, but more are observed for (**4**–**6**). The increased number of vibrations indicates a lowered symmetry of the structure of the AsF_6_^−^ and SbF_6_^−^ anions.

### 2.3 Crystal Structure of [(CH_2_)_3_OCOH]^+^[AsF_6_]^−^ (1)

**Figure 3:**
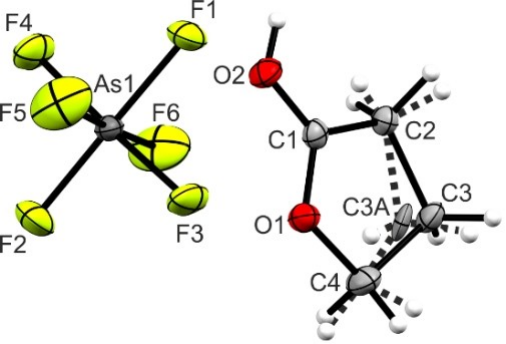
Asymmetric unit of [(CH_2_)_3_OCOH]^+^[AsF_6_]^−^ (**1**) (50 % probability displacement ellipsoids). Dashed lines mark disordered position of the C3 atom (C3A).

The [AsF_6_]^−^ salt of protonated γ‐butyrolactone [(CH_2_)_3_OCOH]^+^[AsF_6_]^−^ (**1**) crystallizes in the monoclinic space group *P*2_1_/*c* with four formula units per unit cell. In Figure 3, the asymmetric unit is shown. Corresponding selected bond lengths and angles are listed in Table 3.

**Table 3:** Selected bond lengths [Å] and angles [°] of [(CH_2_)_3_OCOH]^+^[AsF_6_]^−^ (**1**).

Bond lengths [Å]			
C1−O2	1.280(3)	C4−C3A	1.45(1)
C1−O1	1.273(3)	C3−C2	1.543(8)
O1−C4	1.494(3)	C3A−C2	1.54(2)
C4−C3	1.520(8)	C1−C2	1.471(3)

Bond angles [°]			
O2−C1−C2	128.3(2)	O1−C4−C3	105.6(4)
C1−O1−C4	109.3(2)	C4−C3−C2	103.5(5)
O2−C1−O1	116.0(2)	C4−C3A−C2	107(1)
C2−C1−O1	115.7(2)	C3−C2−C1	102.9(4)

Dihedral angles [°]			
O2−C1−O1−C2	–179.8(4)	O2−C1−C2−C3A	−169.0(7)
O2−C1−O1−C4	177.3(2)	C2−C3−C4−O1	−17.0(5)
O2−C1−C2−C3	171.6(4)	C2−C3A−C4−O1	14(1)

Interatomic contacts [Å]			
O2−H1⋅⋅⋅F1*i*	2.581(2)	C1⋅⋅⋅F3	2.875(3)
C1⋅⋅⋅F4*ii*	2.957(3)

The C3 atom of the cation is disordered, with a 67 % occupancy of the C3 position, whereas position C3A is occupied by 33 %. The bond lengths between C3A and the adjacent carbon atoms (C4−C3A and C3A−C2) are in parts significantly shorter compared to C4−C3 and C3−C2. In the following, only the main species is described in the text, but bond lengths and angles for the other disordered species are given in Table 3. Due to the protonation, the C1−O2 bond is elongated (1.280(3) Å), compared to the neutral compound,^[13]^ whereas the C1−O1 bond length is shortened (1.273(3) Å). These bond lengths are not significantly different and in the range between a formal single (1.43 Å) and double bond (1.19 Å).^[16]^ The C−C bond lengths are in a range between 1.471(3) Å (C1−C2) and 1.543(8) Å (C2−C3). Thus, the C1−C2 bond is significantly shortened compared to γ‐butyrolactone (1.530(1) Å).^[13]^ In consequence of the protonation, the O1−C1−C2 angle is significantly larger (115.7(2)°), compared to the starting material (99.0(3)°).^[13]^ Around the planar lactone moiety (with a dihedral angle of −179.8(4)°), the other angles amount to 116.0(2)° (O1−C1−C2) and 128.3(2)° (O2−C1−C2).

**Figure 4:**
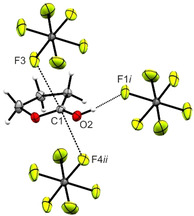
Projection of interatomic contacts in the [(CH_2_)_3_OCOH]^+^[AsF_6_]^−^ (**1**) crystal (50 % probability displacement ellipsoids). Symmetry codes: *i*=1−x, 1−y, −z; *ii*=1+x, y, z.

In Figure 4, a projection of observed interatomic contacts in the [(CH_2_)_3_OCOH]^+^[AsF_6_]^−^ (**1**) crystal is illustrated. A moderate hydrogen bond is observed between the cation and anion O2−H1⋅⋅⋅F1*i* (2.581(2) Å). The strength of hydrogen bonds is classified by *Jeffrey*.^[17]^ Additionally, two interatomic contacts between C1 and F3 (2.875(3) Å) and between C1 and F4*ii* (2.957(3) Å) are found, which are below of the sum of the van der Waals radii (3.17 Å).^[18]^

The bond lengths of the AsF_6_^−^ anion range from 1.691(2) Å (As1−F5) to 1.757(2) Å (As1−F1). The obtained angles are between 88.3(1)° (F3−As1−F1) and 91.9(1)° (F2−As1−F5). For opposite fluorine atoms, an angle of 180° is expected and values from 177.2(1)° (F3−As1−F4) to 179.3(1)° (F1−As1−F2) are observed. All these values are in accordance with reported literature data for AsF_6_^−^ anions.[^19^, ^20^, ^21^]

### 2.4 Crystal Structure of [(CH_2_)_3_NHCOH]^+^[AsF_6_]^−^ (4)

**Figure 5:**
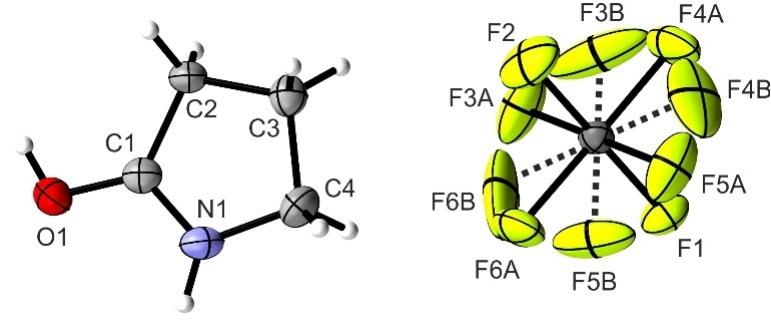
Asymmetric unit of [(CH_2_)_3_NHCOH]^+^[AsF_6_]^−^ (**4**) (50 % probability displacement ellipsoids). Dashed lines mark disordered positions of F3B, F4B, F5B and F6B.

[(CH_2_)_3_NHCOH]^+^[AsF_6_]^−^ (**4**) crystallizes in the orthorhombic space group *P*2_1_2_1_2_1_ with four formula units per unit cell. The asymmetric unit is illustrated in Figure 5. Selected bond lengths and angles of [(CH_2_)_3_NHCOH]^+^[AsF_6_]^‐^ are summarized in Table 4.

**Table 4:** Selected bond lengths [Å] and angles [°] of [(CH_2_)_3_NHCOH]^+^[AsF_6_]^−^ (**4**) with estimated standard deviations in parentheses. Symmetry codes: *i*=−x, −1/2 +y, 1/2 −z; *ii*=−1/2 +z, 1/2 −y, −z; *iii*=x, −1+y, z.

Bond lengths [Å]			
C1−N1	1.283(4)	C4−C3	1.519(6)
C1−O1	1.301(4)	C3−C2	1.529(5)
N1−C4	1.465(5)	C1−C2	1.479(5)

Bond angles [°]			
O1−C1−N1	120.9(3)	C2−C3−C4	106.0(3)
C2−C1−N1	112.3(3)	C3−C4−N1	103.5(3)
O1−C1−C2	126.7(3)	C4−N1−C1	113.7(3)
C1−C2−C3	103.4(3)

Dihedral angles [°]			
O1−C1−N1−C4	179.0(3)	O1−C1−C2−N1	−179.0(6)
O1−C1−C2−C3	174.4(3)	C2−C3−C4−N1	−10.2(4)

Interatomic contacts [Å]			
N1−H5⋅⋅⋅F6A*i*	2.906(6)	O1−H1⋅⋅⋅F1*iii*	2.656(4)
C1⋅⋅⋅F4A*ii*	2.787(7)

**Figure 6:**
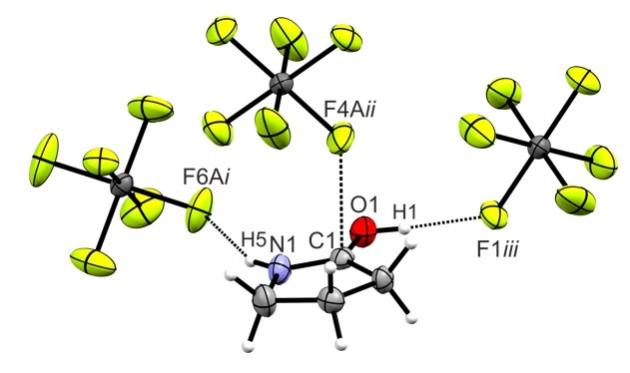
Projection of interatomic contacts in the [(CH_2_)_3_NHCOH]^+^[AsF_6_]^−^ (**4**) crystal (50 % probability displacement ellipsoids). Symmetry codes: *i*=−x, −1/2 +y, 1/2 −z; *ii*=−1/2 +z, 1/2 −y, −z; *iii*=x, −1+y, z.

For the neutral compound γ‐butyrolactam, a lactam‐lactim tautomerism is observed.^[22]^ Therefore, the C1−O1 bond length is, with a value of 1.238(2) Å,^[23]^ in the range between a formal single and double bond.^[16]^ The C1−N1 bond length (1.335(2) Å)^[23]^ is also found to be in between a formal C−N single (1.47 Å) and double bond (1.22 Å).^[16]^ Due to the protonation, the C1−N1 bond length is significantly shortened (1.283(4) Å) and can be considered as a C−N double bond. In contrast, the C1−O1 bond length is elongated (1.301(4) Å), compared to the neutral compound.^[23]^ The N1−C4 and C2−C3 bond lengths nearly are unaffected by the protonation, whereas the C1−C2 and C3−C4 bond lengths are significantly shortened (1.479(5) Å and 1.519(6) Å, respectively). Changes of bond angles are only observed around the planar lactam moiety. The C2−C1−N2 angle is enlarged to 112.3(3)° and the O1−C1−N1 angle is reduced to 120.9(3)°. All other bond angles remain nearly unaffected by the protonation. Figure 6 shows the projection of interatomic contacts in the [(CH_2_)_3_NHCOH]^+^[AsF_6_]^−^ (**4**) crystal.

The hydrogen bonds N1−H5⋅⋅⋅F6A*i* (2.906(6) Å) and O1−H1⋅⋅⋅F1*iii* (2.656(4) Å) are classified as moderate by *Jeffrey*.^[17]^ Another cation‐anion contact is detected between C1 and F4A*ii*. With a value of 2.787(7) Å, this distance is below the sum of the van der Waals radii (3.17 Å).^[18]^

The AsF_6_^−^ anion exhibits a disorder of four fluorine atoms. The A positions are occupied by 61 % and the B positions by 39 %. The bond lengths of the AsF_6_^−^ anion range from 1.675(6) Å to 1.740(2) Å. For neighboring fluorine atoms, values for bond angles between 85.9(2)° (F6A−As1−F2) and 94.2(2)° (F6A−As1−F2) are observed. The bond angles between opposite fluorine atoms nearly measure 180°, as it is expected for *O*_h_ symmetry. All values are in good agreement with data reported in literature.[^19^, ^20^, ^21^]

### 2.5 Theoretical Calculations

**Figure 7:**
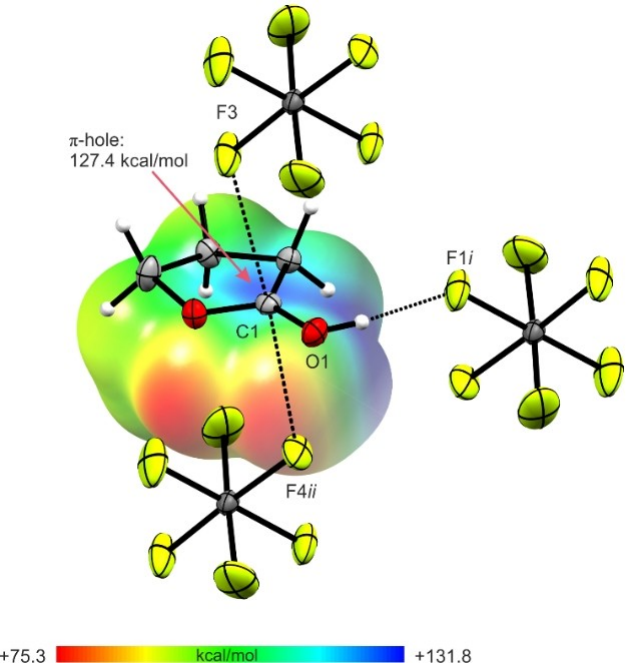
Contacts in the crystal structure (**1**) (50 % probability displacement ellipsoids). Interatomic contacts are drawn as dashed lines. In the background of the cation, the MEP is illustrated with a color range of 75.3 kcal/mol (red) and 131.8 kcal/mol (blue), isoval.=0.0004. Symmetry codes: *i*=1−x, 1−y, −z; *ii*=1+x, y, z.

**Figure 8:**
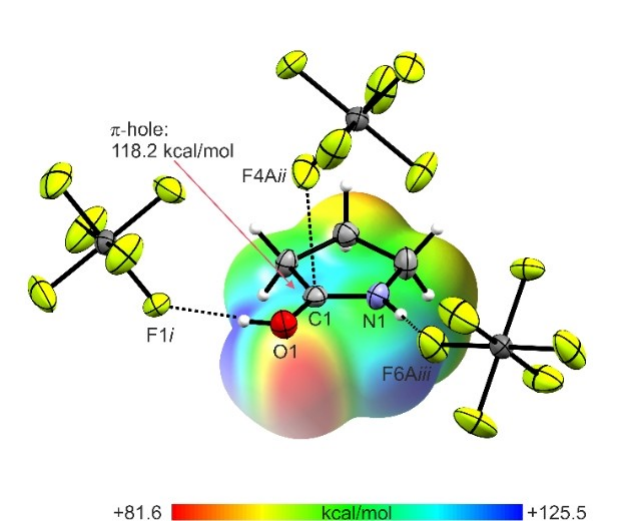
Contacts in the crystal structure (**4**) (50 % probability displacement ellipsoids). Interatomic contacts are drawn as dashed lines. In the background of the cation, the MEP is illustrated with a color range of 81.6 kcal/mol (red) and 125.5 kcal/mol (blue), isoval.=0.0004. Symmetry codes: *i*=x, −1+y, z; *ii*=−1/2 +x, 1/2 −y, −z; *iii*=−x, −1/2 +y, 1/2 −z.

All quantum chemical calculations were carried out on the B3LYP/aug‐cc‐pVTZ level of theory. In both crystal structures C⋅⋅⋅F contacts are observed. Usually, organic cations of hexafluoridometalates only possess hydrogen bonds as interionic interactions. In the case of protonated γ‐butyrolactone and γ‐butyrolactam, we found C⋅⋅⋅F contacts. Such contacts, which differ from hydrogen bonds, have only rarely been observed.[^24^, ^25^, ^26^] To investigate the nature of these contacts, Mapped Electrostatic Potentials (MEP) of the free cations [(CH_2_)_3_OCOH]^+^ and [(CH_2_)_3_NHCOH]^+^ were calculated. The cation‐anion contacts together with the respective MEPs are illustrated in Figures 7 and 8. MEP calculations together with Natural Population Analysis charges (NPA) for the neutral compounds as well as for the protonated species of γ‐butyrolactone (Figure S1) and γ‐butyrolactam (Figure S2) are displayed in the Supporting Information.

Both MEPs show that the electron deficient moiety (region of higher positive potential) of each cation is located in the region of the sp^2^‐hybridized carbon atom. The so‐called π‐hole^[27]^ indicates that the positive charge is located at this atom. This assumption is confirmed by comparing the NPA charges of the neutral compound and the protonated species. For both γ‐butyrolactone and γ‐butyrolactam, the same trend is observed. Due to the protonation, changes of the NPA charges are detected only for the OCO group, respectively the OCN group. For all other atoms NPA charges remain nearly unaffected. Compared to the respective neutral compound, the positive charge of the carbon atom is increased, while the negative charges of the O (or N) atoms are decreased. For electron deficient sp^2^‐hybridized carbon atoms, especially carbonyl groups, investigations on their ability to develop interatomic contacts were performed.[^28^, ^29^] The associated MEP value at the π‐hole of [(CH_2_)_3_OCOH]^+^ (127.4 kcal/mol) is calculated to be more positive compared to the value of [(CH_2_)_3_NHCOH]^+^ (118.2 kcal/mol). The analysis predicts a stronger C⋅⋅⋅F contact in protonated γ‐butyrolactone than in protonated γ‐butyrolactam. This prediction is confirmed by the crystal structure of (**1**), exhibiting the formation of two C⋅⋅⋅F contacts (C1−F3: 2.875(3) Å and C1−F4*ii*: 2.957(3) Å), which are below the sum of the van der Waals radii.^[18]^ In contrast, only one C⋅⋅⋅F contact (C1−F4A*ii*: 2.787(7) Å) is found in (**4**). The more positive MEP value of [(CH_2_)_3_OCOH]^+^ represents the ability to form two contacts, which are therefore slightly weaker than that in (**4**). Summarizing these results, it can be stated that in protonated γ‐butyrolactone, the localization of the positive charge on the sp^2^‐hybridized carbon atom is even stronger than in protonated γ‐butyrolactam.

## 3 Conclusions

In this work, the reaction behaviors of γ‐butyrolactone and γ‐butyrolactam in the superacidic systems *X*F/*M*F_5_ (*X*=H, D; &bk,*M*=As, Sb) were investigated. Only salts of the respective monoprotonated species were obtained and no ring opening reaction was observed. The salts were characterized by Raman and IR spectroscopy. In case of [(CH_2_)_3_OCOH]^+^[AsF_6_]^−^ (**1**) and [(CH_2_)_3_NHCOH]^+^[AsF_6_]^−^ (**4**), single‐crystal X‐ray analyses were performed. In both crystal structures C⋅⋅⋅F contacts between anion and cation, formed from the sp^2^‐hybridized carbon atoms, were observed. In order to investigate the nature of these contacts, Mapped Electrostatic Potentials (MEP) of the cations were calculated. For both cations, regions of positive potential (π‐holes) are located on the sp^2^‐hybridized carbon atoms. The more positive π‐hole MEP value was found for [(CH_2_)_3_OCOH]^+^ compared to [(CH_2_)_3_NHCOH]^+^. Interestingly, this did not lead to the formation of a stronger C⋅⋅⋅F contact in (**1**), but to the formation of two weaker ones. In contrast, a stronger C⋅⋅⋅F contact is observed in (**4**), where the less positive MEP value is calculated for [(CH_2_)_3_NHCOH]^+^.

## Experimental Section

### General

**Caution**! The hydrolysis of AsF_5_, SbF_5_ and the prepared salts (**1**–**6**) might form HF which burns skin and causes irreparable damage. Safety precautions must be taken while using and handling these materials.

### Apparatus and Materials

The reactions were conducted in standard Schlenk technique using a stainless steel vacuum line. FEP/PFA reactors, closed with a stainless steel valve, were used to perform all reactions in superacidic media. The vacuum line, as well as the reactors, were dried with fluorine prior to use. Low temperature Raman spectroscopic investigations were carried out on a Bruker MultiRAM FT‐Raman spectrometer with Nd:YAG laser excitation (λ=1064 cm^−1^) in vacuum at −196 °C. For a measurement, the synthesized compounds were transferred into a cooled glass cell. Low temperature IR spectra were recorded on a Bruker Vertex‐80 V FTIR spectrometer ($\tilde{v}$ =350 to 4000 cm^−1^). A small amount of the synthesized samples was placed on a CsBr single‐crystal plate in a cooled cell for the measurement. The low temperature single‐crystal X‐ray diffractions of [(CH_2_)_3_OCOH]^+^[AsF_6_]^−1^ (**1**) and [(CH_2_)_3_NHCOH]^+^[AsF_6_]^−1^ (**4**) were performed on an Oxford XCalibur 3 diffractometer equipped with a Kappa CCD detector, operating with Mo‐Κα (0.71073 Å) radiation and a Spellman generator (voltage 50 kV, current 40 mA). Data collection was performed at 100 K using the CrystalAlis CCD software^[30]^ and the reduction was carried out using CrysAlis RED software.^[31]^ The structures were solved and refined utilizing SHELXS‐97^[32]^ and SHELXL‐97,^[33]^ belonging to the WinGX software package. Afterwards, the structures were verified by PLATON software.^[34]^ The absorption correction was accomplished by using the SCALE3 ABSPACK multiscan method.^[35]^ Selected data and parameters of the single‐crystal X‐ray structure analyses are summarized in Table S3 for (**1**), and Table S4 for (**4**), respectively (see Supporting Information). Crystallographic data (excluding structure factors) for the structures in this paper were deposited with the Cambridge Crystallographic Data Centre, CCDC, 12 Union Road, Cambridge CB21EZ, UK. Copies of the data can be obtained free of charge on quoting the depository number CCDC‐2013823 for [(CH_2_)_3_OCOH]^+^[AsF_6_]^−^ (**1**) and CCDC‐2013703 for [(CH_2_)_3_NHCOH]^+^[AsF_6_]^−^ (**4**) (Fax: +44‐1223‐336‐033; E‐Mail: deposit@ccdc.cam.ac.uk, http://www.ccdc.cam.ac.uk). The quantum chemical calculations were performed on the B3LYP/aug‐cc‐pVTZ level of theory.^[36]^ For visualization and illustration of the MEP calculations GaussView 6.0 was used.^[37]^

### Synthesis of [(CH_2_)_3_OCOH]^+^[AsF_6_]^−^ (1), [(CH_2_)_3_OCOD]^+^[AsF_6_]^−^ (3), [(CH_2_)_3_NHCOH]^+^[AsF_6_]^−^ (4) and [(CH_2_)_3_NDCOD]^+^[AsF_6_]^−^ (6)

Approximately 2 mL anhydrous hydrogen fluoride (*a*HF), respectively deuterium fluoride (*a*DF), were condensed into a FEP tube‐reactor at −196 °C. Additionally, arsenic pentafluoride (85 mg, 0.5 mmol) was condensed under the same conditions. In order to form the superacidic system, a*X*F (*X*=H, D) and AsF_5_ were warmed to −40 °C and subsequently homogenized. After cooling to −196 °C again, the starting material γ‐butyrolactone, respectively γ‐butyrolactam (43 mg, 0.5 mmol), was added under inert gas atmosphere. The respective reaction mixture was warmed to −40 °C and homogenized until the salt was completely dissolved. Excess *aX*F was removed at −78 °C overnight in a dynamic vacuum. For crystallization of compounds (**1**) and (**4**), the reactors were left in an ethanol bath at −50 °C until the salts recrystallized. Compounds [(CH_2_)_3_OCOH]^+^[AsF_6_]^−^ (**1**) and [(CH_2_)_3_OCOD]^+^[AsF_6_]^−^ (**3**) were obtained as colorless solids, which decompose at −30 °C. Compounds [(CH_2_)_3_NHCOH]^+^[AsF_6_]^−^ (**4**) and [(CH_2_)_3_NDCOD]^+^[AsF_6_]^−^ (**6**), likewise colorless solids, are stable up to room temperature.

### Synthesis of [(CH_2_)_3_OCOH]^+^[SbF_6_]^−^ (2) and [(CH_2_)_3_NHCOH]^+^[SbF_6_]^−^ (5)

First, antimony pentafluoride (108 mg, 0.5 mmol) was condensed into a FEP tube‐reactor at −196 °C. Subsequently, anhydrous hydrogen fluoride (2 mL) was condensed into the reactor under the same conditions. To form the superacidic system, both compounds were allowed to warm to −40 °C and homogenized. After cooling to −196 °C again, the respective starting material, γ‐butyrolactone (43 mg, 0.5 mmol) or γ‐butyrolactam (43 mg, 0.5 mmol), was added under inert gas atmosphere. For the desired protonation, the mixture was warmed to −40 °C and homogenized until the respective salts were completely dissolved. Excess *a*HF was removed at −78 °C in a dynamic vacuum overnight. [(CH_2_)_3_OCOH]^+^[SbF_6_]^−^ (**2**) and [(CH_2_)_3_NHCOH]^+^[SbF_6_]^−^ (**5**) were obtained as colorless solids, with a decomposition temperature of −30 °C (**2**), while compound (**5**) is stable up to room temperature.

## Conflict of interest

The authors declare no conflict of interest.

## Supporting information

As a service to our authors and readers, this journal provides supporting information supplied by the authors. Such materials are peer reviewed and may be re‐organized for online delivery, but are not copy‐edited or typeset. Technical support issues arising from supporting information (other than missing files) should be addressed to the authors.

Supplementary
