# Supplementary material for: Protonation of γ‐Butyrolactone and γ‐Butyrolactam
Source: ChemistryOpen. 2020 Dec 17;10(1):8–15. doi: 10.1002/open.202000220 (PMC7780814; doi:10.1002/open.202000220)
Supplement: Supplementary file 1 — Supplementary [file OPEN-10-8-s001.pdf]

ChemistryOpen

Supporting Information

Protonation of γ-Butyrolactone and γ-Butyrolactam

Stefanie Beck, Michael Feller, Laura Spies, Kai J. Dietrich, Christoph Jessen, Karin Stierstorfer, and Andreas J. Kornath*

Supporting Information

Table S1. Experimental vibrational frequencies [cm^{-1}] of (**1**), (**2**) and (**3**), and calculated vibrational frequencies [cm^{-1}] of $[(CH_2)_3OCOH]^+$ and $[(CH_2)_3OCOD]^+$.

Table S2. Experimental vibrational frequencies [cm^{-1}] of (**4**), (**5**) and (**6**), and calculated vibrational frequencies [cm^{-1}] of $[(CH_2)_3NHCOH]^+$ and $[(CH_2)_3NDCOD]^+$.

Table S3. Crystal data and structure refinement for $[(CH_2)_3OCOH]^+[AsF_6]^-$ (**1**).

Table S4. Crystal data and structure refinement for $[(CH_2)_3NHCOH]^+[AsF_6]^-$ (**4**).

Figure S1. Electrostatic potential isosurfaces and the NPA charges have been calculated for $C_4H_6O_2$ (left) with a color range of –0.055 a.u. (red) to 0.036 a.u. (blue), isoval = 0.0004; and for $[C_4H_7O_2]^+$ (right) with a color range of 0.12 a.u. (red) and 0.21 a.u. (blue), isoval. = 0.0004.

Figure S2. Electrostatic potential isosurfaces and the NPA charges have been calculated for C_4H_7NO (left) with a color range of –0.061 a.u. (red) to 0.045 a.u. (blue), isoval = 0.0004; and for $[C_4H_8NO]^+$ (right) with a color range of 0.13 a.u. (red) and 0.20 a.u. (blue), isoval. = 0.0004.

Table S1. Experimental vibrational frequencies [cm^{-1}] of (**1**), (**2**) and (**3**), and calculated vibrational frequencies [cm^{-1}] of [$(CH_2)_3OCOH$]$^+$ and [$(CH_2)_3OCOD$]$^+$.

**[$(CH_2)_3OCOH$]$^+$[AsF_6]$^-$ (1)**		**[$(CH_2)_3OCOH$]$^+$[SbF_6]$^-$ (2)**		**[$(CH_2)_2OCOD$]$^+$[AsF_6]$^-$ (3)**		**[$(CH_2)_3OCOH$]$^+$**	**[$(CH_2)_3OCOD$]$^+$**	**Assignment[b]**
**IR**	**Raman**	**IR**	**Raman**	**IR**	**Raman**	**Calc. (IR/Ra)[a]**	**Calc. (IR/Ra)[a]**	
				3518 (vw, br)				ν(OH)
3528 (vw, br)		3460 (vw, br)		2307 (m)	2293 (14)	3724 (256/61)	2714 (148/29)	ν(O*X*)
	3078 (22)	3078 (m)	3077 (10)	3072 (m)	3078 (34)	3181 (0.4/57)	3181 (0.4/57)	$\nu_{as}(CH_2)$
	3040 (28)				3040 (42)	3143 (0.2/59)	3143 (0.2/59)	$\nu_{as}(CH_2)$
	3022 (44)		3024 (26)		3023 (62)	3108 (1/114)	3108 (1/114)	$\nu_{s}(CH_2)$
2995 (m)	3004 (60)	3005 (m)	3004 (30)	3001 (m)	3004 (89)	3102 (3/77)	3102 (3/77)	$\nu_{as}(CH_2)$
2930 (m)	2946 (42)	2951 (m)	2950 (31)	2945 (m)	2945 (61)	3089 (2/84)	3089 (2/84)	$\nu_{s}(CH_2)$
	2912 (13)		2905 (6)		2909 (19)	3030 (12/106)	3030 (12/107)	$\nu_{s}(CH_2)$
		2833 (w)						?
		2544 (vw)		2511 (w)				?
		2457 (vw)		2422 (w)				?
				1909 (w)				?
1684 (s)	1613 (7)	1616 (m)	1637 (5)	1607 (s)	1619 (12)	1650 (305/0.7)	1635 (383/0.5)	ν(CO)
	1523 (24)	1522 (m)	1526 (14)	1510 (m)	1510 (27)	1523 (161/4)	1515 (74/8)	ν(CO)
1487 (m)	1476 (18)	1481 (w, sh)	1471 (12)	1524 (m)		1518 (33/6)	1521 (64/3)	$\delta(CH_2)$
1464 (m)	1463 (29)	1460 (w)		1460 (m)	1462 (29)	1502 (13/6)	1500 (17/6)	$\delta(CH_2)$
	1408 (23)		1407 (12)	1404 (m)	1406 (28)	1446 (30/8)	1445 (32/8)	$\delta(CH_2)$
1396 (m)	1397 (17)	1400 (m)	1396 (11)			1371 (2/0.5)	1369 (3/0.8)	$\omega(CH_2)$
		1342 (m)		1337 (m)	1338 (11)			?
1319 (m)		1311 (m)				1340 (30/0.7)	1333 (39/2)	$\omega(CH_2)$
1284 (m)	1286 (10)	1283 (m)	1288 (8)	1298 (m)	1304 (13)	1310 (8/1)	1307 (3/0.8)	$\omega(CH_2)$
1244 (m)	1232 (40)	1227 (m)	1231 (23)	1229 (m)	1231 (43)	1245 (7/5)	1245 (4/5)	$\tau(CH_2)$
1213 (s)	1204 (13)	1205 (m)	1206 (8)		1217 (13)	1235 (65/2)	1231 (19/2)	$\tau(CH_2)$
	1191 (7)	1192 (m)	1194 (6)	1192 (m)	1191 (9)	1207 (20/0.3)	1206 (8/0.4)	$\tau(CH_2)$
1088 (m)	1089 (5)	1084 (w)	1085 (5)	912 (m)	916 (13)	1178 (199/7)	863 (12/1)	δ(CO*X*)
1036 (m)	1038 (25)	1036 (w)	1038 (16)	1034 (m)	1038 (28)	1104 (2/0.2)	1104 (0.6/0.3)	$\rho(CH_2)$
989 (m)		1012 (w)		960 (m)	961 (7)	1029 (3/3)	1029 (6/3)	ν(CC)
955 (m)	959 (33)	959 (m)	961 (24)	1001 (s)	1000 (21)	949 (5/4)	993 (107/6)	ν(CO)
932 (m)	938 (23)	937 (w)	939 (13)	939(m)	941 (35)	934 (1/3)	934 (2/4)	ν(CC)
						896 (7/4)	895 (6/4)	$\rho(CH_2)$
881 (m)	877 (38)	876 (m)	878 (28)	876 (m)	875 (33)	881 (17/6)	889 (45/5)	ring breathing
822 (m)		820 (m)		834 (m)				?
806 (m)	800 (13)		793 (8)	800 (m)	799 (15)	796 (11/2)	796 (10/2)	δ(COC)
	723 (6)			729 (s, sh)	723 (8)	684 (10/3)	682 (9/3)	δ(CCC)
	683 (65)		674 (8)	630 (s)	630 (23)	639 (29/3)	626 (10/5)	δ(CCC)
644 (w)	632 (21)	639 (vs)	633 (50)		450 (30)	611 (77/3)	443 (57/0.2)	$\delta(COX)_{oop}$

515 (m)	525 (9)	525 (s)	524 (3)	523 (m)	523 (13)	517 (25/0.5)	535 (1/0.7)	γ(CCOO)
469 (w)	471 (17)	467 (s)	473 (7)			435 (13/0.8)	411 (13/0.8)	δ(OCO)
	238 (6)		234 (2)			218 (5/0.2)	216 (6/0.1)	skeletal vibration
	154 (12)		137 (8)			137 (2/0.3)	137 (2/0.3)	skeletal vibration
	120 (30)							?
698 (vs)	704 (93)	658 (vs)	691 (16)	675 (vs)	704 (97)			$[MF_6]^-$
536 (m)	673 (100)	584 (m)	661 (100)	696 (vs)	682 (56)			$[MF_6]^-$
392 (m)	589 (18)	550 (s)	642 (53)	540 (s)	673 (100)			$[MF_6]^-$
	545 (15)		587 (8)	447 (w)	589 (20)			$[MF_6]^-$
	403 (6)		297 (12)		541 (17)			$[MF_6]^-$
	375 (44)		285 (37)		375 (46)			$[MF_6]^-$
	368 (49)				367 (53)			$[MF_6]^-$

[a] Calculated on the B3LYP/aug-cc-pVTZ level of theory. IR intensity in km/mol and Raman intensity in Å^4/u. Abbreviations for IR intensities: v = very, s = strong, m = medium, w = weak. Experimental Raman activities are stated to a scale of 1 to 100. [b] *X* = H, D.

Table S2. Experimental vibrational frequencies [cm^{-1}] of (**4**), (**5**) and (**6**), and calculated vibrational frequencies [cm^{-1}] of $[(CH_2)_3NHCOH]^+$ and $[(CH_2)_3NDCOD]^+$.

**$[(CH_2)_3NHCOH]^+[AsF_6]^-$ (4)**		**$[(CH_2)_3NHCOH]^+[SbF_6]^-$ (5)**		**$[(CH_2)_2NDCOD]^+[AsF_6]^-$ (6)**		**$[(CH_2)_3NHCOH]^+$**	**$[(CH_2)_3NDCOD]^+$**	**Assignment[b]**
**IR**	**Raman**	**IR**	**Raman**	**IR**	**Raman**	**Calc. (IR/Ra)[a]**	**Calc. (IR/Ra)[a]**	
				3391 (w)				ν(OH)
				3358 (w)	3334 (2)			ν(NH)
3238 (w)		3242 (vs)		2492 (w)	2494 (18)	3750 (235/67)	2732 (137/33)	ν(O*X*)
					2477 (19)			?
3364 (w)	3369 (3)	3358 (s)	3359 (4)	2430 (w)	2445 (16)	3553 (139/82)	2609 (89/37)	ν(N*X*)
					2402 (12)			?
3024 (vw)	3026 (17)	3059 (w)	3033 (11)	3024 (vw)	3028 (36)	3148 (0.3/58)	3148 (0.3/58)	$\nu_{as}(CH_2)$
	3015 (sh)				3015 (43)	3131 (0.4/78)	3131 (0.4/78)	$\nu_{as}(CH_2)$
2982 (vw)	2991 (27)	2995 (vw)	3000 (25)	2982 (vw)	2995 (58)	3098 (0.04/106)	3098 (0.06/106)	$\nu_{as}(CH_2)$
2947 (vw)	2945 (33)		2944 (27)	2949 (vw)	2947 (40)	3089 (4/48)	3089 (4/48)	$\nu_s(CH_2)$
	2931 (sh)	2918 (vw)	2919 (15)		2923 (20)	3074 (3/139)	3074 (3/139)	$\nu_s(CH_2)$
	2907 (sh)				2901 (11)	3037 (7/121)	3037 (7/122)	$\nu_s(CH_2)$
				2748 (vw)				?
				1807 (vw)				?
1711 (m)	1716 (16)	1711 (m)	1711 (14)	1691 (m)	1691 (17)	1729 (251/3)	1703 (278/5)	ν(CN)
			1498 (6)	1485 (m)	1482 (28)	1525 (2/8)	1524 (0.9/9)	$\delta(CH_2)$
1491 (m)	1489 (26)	1489 (m)	1485 (15)	1472 (m)	1469 (19)	1511 (108/4)	1508 (30/5)	$\delta(CH_2)$
1462 (w)	1461 (16)	1464 (vw)	1464 (14)	1462 (m)	1456 (24)	1500 (103/7)	1474 (140/5)	ν(CO)
1419 (w)	1421 (22)	1423 (w)	1421 (17)	1417 (m)	1417 (39)	1460 (207/10)	1460 (21/10)	$\delta(CH_2)$
1390 (w)	1393 (4)	1393 (vw)		1186 (w)	1172 (7)	1404 (3/0.7)	1176 (6/2)	δ(CN*X*)

				1375 (w)	1337 (7)	1351 (6/0.3)	1368 (7/1)	$\omega(CH_2)$
1319 (w)	1323 (10)	1317 (w)	1323 (8)	1321 (m)	1325 (6)	1343 (23/3)	1346 (19/2)	$\omega(CH_2)$
1307 (w)		1296 (vw)	1288 (5)			1312 (5/1)	1311 (3/0.9)	$\omega(CH_2)$
1261 (m)	1263 (7)	1256 (vw)	1259 (6)	1279 (vw)	1279 (5)	1254 (2/5)	1263 (11/1)	$\tau(CH_2)$
1229 (w)	1230 (24)	1230 (vw)	1233 (17)	1231 (w)	1233 (23)	1235 (21/2)	1253 (3/4)	$\tau(CH_2)$
			1204 (6)			1208 (10/0.5)	1207 (4/0.4)	$\tau(CH_2)$
1186 (w)	1189 (5)		1189 (4)	954 (w, sh)	956 (13)	1198 (138/8)	915 (28/9)	$\delta(COX)$
1078 (vw)	1079 (2)		1083 (4)	1078 (vw)	1078 (4)	1099 (0.4/0.2)	1098 (0.09/0.2)	$\rho(CH_2)$
1049 (w)	1052 (10)		1048 (9)	1055 (m)	1056 (6)	1042 (2/2)	1032 (0.3/3)	ν(CN)
				1011 (s)	1009 (8)			?
982 (m)	983 (20)	982 (vw)	982 (20)	943 (m)	938 (9)	971 (10/3)	938 (11/5)	ν(CC)
926 (w)	924 (sh)	928 (vw)	928 (5)	775 (s)	778 (6)	929 (3/0.2)	809 (6/0.4)	ν(CC)
				920 (m)		905 (3/2)	905 (3/2)	$\rho(CH_2)$
895 (w)	897 (73)	903 (vw)	902 (58)	895 (m)	895 (79)	896 (5/17)	889 (52/8)	ring breathing
824 (vw)		820 (vw)	815 (4)	839 (vw)	842 (14)	817 (3/0.9)	831 (3/4)	δ(CCC)
783 (w)		777 (vw)	782 (4)	476 (m)	476 (10)	761 (65/0.8)	463 (25/0.4)	$\delta(CNX)_{oop}$
		708 (vw)	694 (8)	584 (m)	583 (23)	702 (6/4)	694 (3/4)	δ(CCN)
620 (s)	620 (8)	617 (vw, sh)	620 (10)	619 (s, sh)	608 (11)	619 (9/3)	598 (9/3)	δ(CCC)
555 (s)	554 (12)	554 (vw)	556 (11)			552 (132/0.7)	391 (66/0.2)	$\delta(COX)_{oop}$
517 (m)	518 (9)	515 (vw)	517 (11)	559 (s)	560 (16)	497 (24/0.6)	665 (21/1)	γ(CCON)
453 (w)	454 (13)		447 (19)			420 (9/1)	392 (10/0.9)	δ(OCN)
	240 (4)		248 (9)		237 (9)	224 (0.3/0.2)	219 (0.4/0.1)	skeletal vibration
						122 (0.9/0.3)	121 (0.8/0.3)	skeletal vibration
704 (vs)	705 (35)	667 (s)	671 (18)		700 (63)			$[MF_6]^-$
671 (vs)	679 (100)	642 (m)	658 (100)		689 (80)			$[MF_6]^-$
579 (s)	580 (18)	563 (vw)	642 (34)		673 (100)			$[MF_6]^-$
	561 (12)		579 (14)		430 (17)			$[MF_6]^-$
	371 (43)		565 (12)		370 (61)			$[MF_6]^-$
			280 (42)					$[MF_6]^-$

[a] Calculated on the B3LYP/aug-cc-pVTZ level of theory. IR intensity in km/mol and Raman intensity in $Å^4/u$. Abbreviations for IR intensities: v = very, s = strong, m = medium, w = weak. Experimental Raman activities are stated to a scale of 1 to 100. [b] *X* = H, D.

Table S3. Crystal data and structure refinement for $[(CH_2)_3OCOH]^+[AsF_6]^-$ (**1**).

	$[(CH_2)_3OCOH]^+[AsF_6]^-$ (**1**)
Empirical formular	C4 H7 F6 O2 As
M_r	276.02
Crystal system	monoclinic
Space group	$P2_1/c$
a [Å]	8.2327(5)
b [Å]	9.7948(4)
c [Å]	10.8507(8)
α [°]	90
β [°]	109.891(6)
γ [°]	90
V [$Å^3$]	822.78(9)
Z	4
ρ_{calcd} [gcm^{-3}]	2.228
μ[mm^{-1}]	4.203
$\lambda_{Mo K\alpha}$	0.71073
F(000)	536
T[K]	100(2)
hkl range	–8:10; –11:12;–13:14
refl. measured	3644
refl. unique	1883
R_{int}	0.0187
parameters	132
R(F)/*w*R(F^2)[a)]	0.0317/0.0680
weighting scheme[b)]	0.0356/0.2308
S(GoF)[c)]	1.058
residual density [$eÅ^{-3}$]	0.548/ –0.517
devide type	Oxford XCalibur
solution/refinement	SHELXS-97
CCDC	2013823

a) $R_1 = \Sigma||F_o|-|F_c||/\Sigma|F_o|$; b) $wR_2 = [\Sigma[w(F_o^2-F_c^2)^2]/\Sigma[w(F_o)^2]]^{1/2}$; $w = [\sigma_c^2(F_o^2)+(xP)^2+yP]^{-1}$; $P = (F_o^2+2F_c^2)/3$ c) GoF = $\{\Sigma[w(F_o^2-F_c^2)^2]/(n-p)\}^{1/2}$ (*n* = number of reflexions; p = total number of parameters).

Table S4. Crystal data and structure refinement for $[(CH_2)_3NHCOH]^+[AsF_6]^-$ (**4**).

	$[(CH_2)_3NHCOH]^+[AsF_6]^-$ (**4**)
Empirical formular	C4 H8 F6 N O As
M_r	275.03
Crystal system	orthorhombic
Space group	$P2_12_12_1$
a [Å]	7.7533(3)
b [Å]	10.1067(4)
c [Å]	10.9203(5)
α [°]	90
β [°]	90
γ [°]	90
V [$Å^3$]	855.72(6)
Z	4
ρ_{calcd} [gcm^{-3}]	2.135
μ[mm^{-1}]	4.035
$\lambda_{Mo\kappa\alpha}$	0.71073
F(000)	536
T[K]	109(2)
hkl range	–11:11; –11:15;–16:16
refl. measured	9417
refl. unique	2827
R_{int}	0.0345
parameters	162
R(F)/*w*R(F^2)[a)]	0.0392/0.0679
weighting scheme[b)]	0.0318/0.0614
x(Flack)	0.022(16)/ 0.0679
S(GoF)[c)]	1.044
residual density [$eÅ^{-3}$]	0.482/-0.322
devide type	Oxford XCalibur
solution/refinement	SHELXS-97
CCDC	2013703

a) $R_1 = \Sigma||F_o|-|F_c||/\Sigma|F_o|$; b) $wR_2 = [\Sigma[w(F_o^2-F_c^2)^2]/\Sigma[w(F_o)^2]]^{1/2}$; $w = [\sigma_c^2(F_o^2)+(xP)^2+yP]^{-1}$; $P = (F_o^2+2F_c^2)/3$ c) GoF = $\{\Sigma[w(F_o^2-F_c^2)^2]/(n-p)\}^{1/2}$ (*n* = number of reflexions; p = total number of parameters).

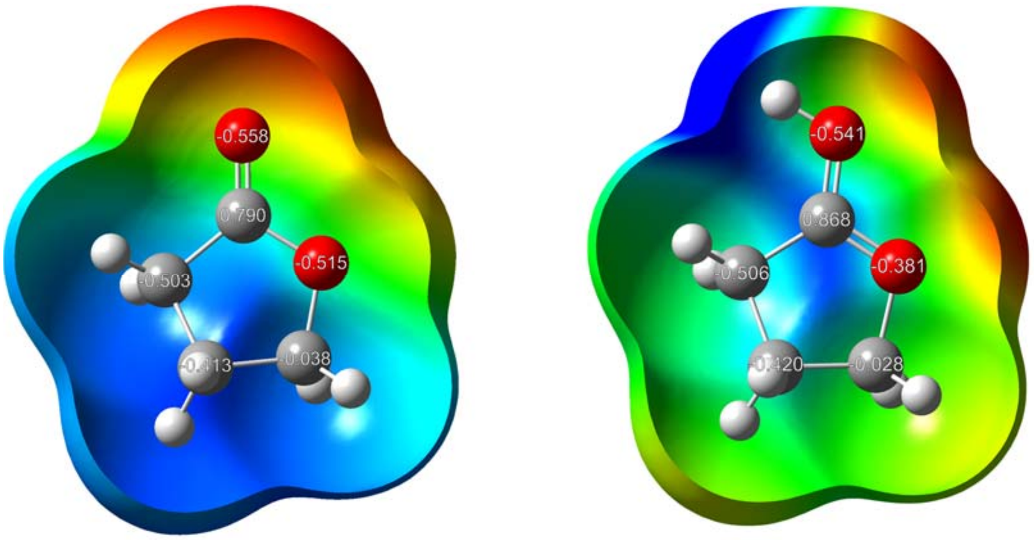

Figure S1. Electrostatic potential isosurfaces and the NPA charges have been calculated for $C_4H_6O_2$ (left) with a color range of –0.055 a.u. (red) to 0.036 a.u. (blue), isoval = 0.0004; and for $[C_4H_7O_2]^+$ (right) with a color range of 0.12 a.u. (red) and 0.21 a.u. (blue), isoval. = 0.0004.

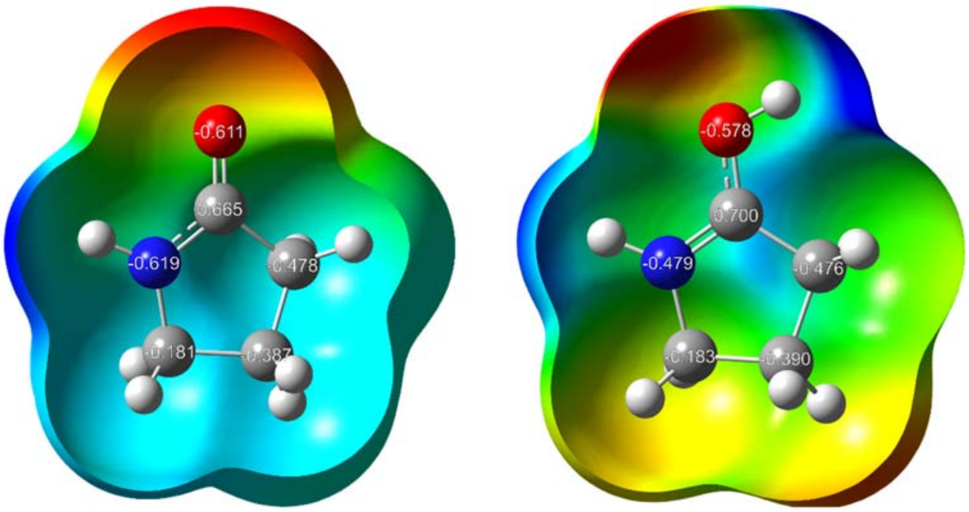

Figure S2. Electrostatic potential isosurfaces and the NPA charges have been calculated for C_4H_7NO (left) with a color range of –0.061 a.u. (red) to 0.045 a.u. (blue), isoval = 0.0004; and for $[C_4H_8NO]^+$ (right) with a color range of 0.13 a.u. (red) and 0.20 a.u. (blue), isoval. = 0.0004.
